# Detection of ‘*Candidatus* Phytoplasma solani’ in roots from Bois noir symptomatic and recovered grapevines

**DOI:** 10.1038/s41598-018-38135-9

**Published:** 2019-02-14

**Authors:** Lucia Landi, Sergio Murolo, Gianfranco Romanazzi

**Affiliations:** Department of Agricultural, Food and Environmental Sciences, Marche Polytechnic University, Via Brecce Bianche, I-60131 Ancona, Italy

## Abstract

‘*Candidatus* Phytoplasma solani’ is the causal agent of Bois noir (BN) in grapevine (*Vitis vinifera*). It is usually detected in leaves, where typical disease symptoms are seen. However, little information is available on the presence of this phytoplasma in grapevine roots. Here, we investigated ‘*Ca*. P. solani’ in roots collected from 28 symptomatic, 27 recovered and eight asymptomatic grapevine plants. Protocols based on high-resolution melting (HRM) combined with real-time quantitative PCR (qPCR-HRM) and nested-qPCR-HRM were developed to identify ‘*Ca*. P. solani’ *tuf*-type variants with single nucleotide polymorphisms. In all, 21.4% of roots from symptomatic plants were positive to ‘*Ca*. P. solani’ using qPCR-HRM, and 60.7% with nested-qPCR HRM. Also, 7.4% of roots from recovered plants were positive using qPCR-HRM, which reached 44.4% using nested-qPCR HRM. These analyses identified *tuf*-type b1 on 88.2% of the positive samples from symptomatic grapevines, and 66.6% from recovered grapevines, with all other samples identified as *tuf*-type a. This study reports the presence of ‘*Ca*. P. solani’ in the roots of both symptomatic and recovered grapevines. These qPCR-HRM and nested-qPCR-HRM protocols can be applied to increase the sensitivity of detection of, and to simplify and speed up the screening for, ‘*Ca*. P. solani’ *tuf*-types.

## Introduction

Grapevine yellows are diseases that can have detrimental effects upon grapevine yields, in terms of both quantity and quality^1,2^. Bois noir (BN) is the most recurrent grapevine yellows phytoplasma disease, and it has been recorded all over Europe, the Mediterranean basin, and in the Middle East^3–5^. BN is caused by ‘*Candidatus* (*Ca*.) Phytoplasma (P.) solani’^6^, which belongs to the stolbur phytoplasma group (16SrXII subgroup A)^7^. The planthopper *Hyalesthes obsoletus* Signoret is known to be the main vector for transmission of ‘*Ca*. P. solani’ in many countries^8^, although several other vectors or potential vectors might be involved^5,9^.

The optimal period for diagnosis of ‘*Ca*. P. solani’ in grapevine leaves in the northern hemisphere is generally from June to September. This is prior to harvest, which for the Chardonnay cultivar is expected from the mid of August to the beginning of September^2^. However, it has been demonstrated that *Candidatus* Phytoplasma australiense’ (16SrXII-B) and Tomato big bud phytoplasma (16SrII-D), associated with Australian Grapevine Yellows^10^, and also ‘*Ca*. P. solani’^11^, have been detected in trunk, cordon, shoots, and roots of phytoplasma-affected grapevines. Furthermore, the presence of ‘*Ca*. P. solani’ has been recorded for the roots of herbaceous plant hosts of *H*. *obsoletus* vectors^12^.

An intriguing aspect of the epidemiology of BN is the process of ‘recovery’, which is the spontaneous disappearance of BN symptoms from previously symptomatic plants^13,14^. In such recovered grapevines, attempts to detect phytoplasma in the canopy have usually failed^15^. However, Hren *et al*.^16^ reported weak amplicons associated with the presence of Flavescence dorée (FD) phytoplasma in one out of six Barbera grapevines that had recovered from FD disease, another important grapevine yellows disease in Europe. Also, in a few cases, phytoplasma DNA has been reported for asymptomatic grapevines^10^. Thus, as reported in various studies, recovered plants are generally not colonised by phytoplasma in the canopy^17,18^.

In apple and pear plants affected by apple proliferation and pear decline, respectively, the degenerated sieve tubes seen from late autumn are in almost all cases eliminated in the aerial parts during winter. Instead, they persist in the roots, where there are intact sieve tubes throughout the year. From the roots, both of these pathogens can recolonise the aerial parts of the plants in spring, when new phloem is formed^19^.

One preliminary study showed that ‘*Ca*. P. solani’ can be detected in grapevine roots of both symptomatic and recovered plants using a nested real-time (RT)-TaqMan PCR test, and it was suggested that the phytoplasma might persist^20^. However, there is little other information available relating to the location and persistence of ‘*Ca*. P. solani’ in grapevine roots. This appears to be because their phytoplasma titre is very low, or because of the presence of inhibitors that affect the molecular tools. At present, however the detection of ‘*Ca*. P. solani’ in grapevine is usually carried out by molecular approaches, starting with the extraction of DNA from leaf samples that are collected from different parts of the canopy of symptomatic plants. This is followed by molecular detection using conventional and/or quantitative RT-(q)PCR for detection of the phytoplasma 16SrRNA gene^21–24^. Restriction fragment length polymorphism (RFLP) of the 16S rRNA gene that is mainly used for routine molecular identification of the phytoplasma species^7^. Molecular characterisation is based on multilocus sequence analysis carried out on several genes, to more accurately identify the phytoplasma strains. In particular, the *secY*, *vmp*1 and *stamp* genes have been associated with more precise characterisation of the genetic diversity of ‘*Ca*. P. solani’^4,25–28^, while the *tuf* gene is used to analyse the natural epidemic cycles of stolbur phytoplasma^29–31^.

High-resolution melting (HRM) can be useful for detection of genetic variants^32,33^. This technology detects changes in fluorescence during the melting of double-stranded DNA during determination of the dissociation curves of specific PCR amplicons that are produced using RT-PCR instrumentation that has precise temperature-ramp control (i.e., *ca*. 0.01 °C to 0.2 °C)^34,35^. HRM has considerable advantages over conventional methods, as it is carried out in a closed tube and represents a very rapid and cost-effective gene-scanning method, with no sample processing required after PCR amplification^35^. HRM can detect single nucleotide insertion and deletion polymorphisms, insertion–deletion polymorphisms, and simple sequence repeat markers, and avoids the need to also sequence the wild-type DNA^36^.

The goal of the present study was to analyse roots from symptomatic and recovered grapevines for the presence of ‘*Ca*. P. solani’. To achieve this, a specific HRM assay was developed to discriminate *tuf*-type variants using qPCR-HRM and nested-qPCR-HRM assays.

## Results

### Set-up of qPCR-HRM and nested-qPCR HMR for ‘*Ca*. P. solani’ detection

Different trials to optimise the qPCR-HMR started from the different matrices (i.e., leaves, roots) spiked with serial dilutions of ‘*Ca* P. solani’ PCR *tuf* fragments. These revealed that, related to DNA from roots, at concentrations >25 ng/reaction, the PCR was inhibited, while this not was shown with DNA from leaves until 100 ng/reaction. For the leaves, PCR inhibition was observed at 500 ng/reaction (Table 1). In particular, for ‘*Ca*. P. solani’ detection in root samples, 5 ng/reaction DNA target provided the appropriate dilution (data not shown). No amplification was observed in the negative controls. The limit of quantification (LOQ) of PCR *tuf* fragments corresponded to around 40 copies/reaction of *tuf*-PCR fragment for both purified PCR fragments alone or combined with root and leaf DNA (Table 1). All of the standard curves performed according to samples artificially spiked with ‘*Ca* P. solani’ PCR *tuf* fragments, P7 and 19–25 calibrators, and Sy5/4-infected samples indicated that the assay was operating at 100% ± 10% efficiency, except for the Sy5/4 roots, which showed poor mean efficiency (135.2%) (Tables 1 and 2). A similar limit of detection (LOD) was observed among the samples tested, which ranged from mean Cq of 35.28 to 37.19 (Tables 1 and 2). The Cq values of all of the samples confirmed the reproducibility within a low coefficient of variation (CV) of between 0.36%–3.8% (CV <25%)^37^ (Tables 1 and 2). For the nested qPCR-HRM set-up, the optimal cycle number for the first PCR was 35, because the *C*_*q*_ showed an elevated concentration that remained proportional to the differences between all of the starting quantities (Table 3). For the nested-qPCR-HRM assays, the PCR product diluted at 1/200 showed the characteristic melting temperature peak for all samples analysed. Therefore, 35 cycles was adopted as the optimal cycle number for the PCR.

**Table 1:** The qPCR-HRM inhibitors and limits of quantification estimated by standard curve performance according to ‘*Candidatus* Phytoplasma solani’ *tuf* gene detection for: PCR fragment obtained in qPCR-HRM from Periwinkle infected by ‘*Candidatus* Phytoplasma solani’ for P7 and 19–25 isolates; different concentration of grapevine root genomic DNA (500, 100, 75, 25 and 5 ng/qPCR-HRM reaction) and leaf genomic DNA (500, 100 and 5 ng/qPCR-HRM reaction) spiked with serial dilutions of P7 *tuf* PCR fragment of ‘*Ca*. P. solani’.

	*tuf* PCR fragments									
	From calibrators		From P7 calibrator with DNA from roots					From P7 calibrators with DNA from leaves		
	19–25	P7	500	100	75	25	5	500	100	5
**Concentration of** ***tuf*** **PCR fragment (copies/reaction)**	***Cq*** **mean** ± **SD (CV%)**^**(a)**^									
4.01 × 10^5^	23.06 ± 0.24 (1.01)	23.59 ± 0.16 (0.67)	na	na	31.1*	23.21 ± 0.42 (1.8)	23.16 ± 0.71 (3.06)	na	23.07 ± 0.4 (1.73)	21.95 ± 0.34 (1.54)
4.01 × 10^4^	26.48 ± 0.2 (0.75)	27.09 ± 0.25 (0.92)	na	na	na	26.51 ± 0.38 (1.43)	26.45 ± 0.44 (1.66)	na	26.41 ± 0.2 (0.57)	25.12 ± 0.31 (1.23)
4.01 × 10^3^	29.81 ± 0.3 (1.0)	30.55 ± 0.29 (0.94)	na	na	30.4*	29.99 ± 0.31 (1.03)	29.71 ± 0.22 (0.74)	na	29.69 ± 0.47 (1.58)	28.13 ± 0.38 (1.35)
4.01 × 10^2^	33.45 ± 0.31 (0.92)	33.55 ± 1.3 (3.87)	na	na	na	33.11 ± 0.57 (1.72)	33.15 ± 1.05 (3.16)	na	33.30 ± 0.16 (0.48)	31.89 ± 0.82 (2.57)
4.01 × 10^1^	36.68 ± 0.46 (1.25)	36.50 ± 1.4 (3.8)	na	na	na	36.39 ± 1.01 (2.77)	37.19 ± 0.99 (2.66)	na	36.71 ± 1.31 (3.6)	35.69 ± 0.47 (1.31)
4.01	na	37.3*	na	na	na	na	na	na	na	na
**Statistics of standard curve performance, mean** **±** **SD**										
Slope	−3.409 ± 0.016	−3.365 ± 0.024	nd	nd	nd	−3.299 ± 0.027	−3.412 ± 0.040	nd	−3.423 ± 0.095	−3.428 ± 0.038
Efficiency	96.46 ± 0.64	98.20 ± 0.98	nd	nd	nd	101.0 ± 1.27	96.4 ± 1.53	nd	95.9 ± 3.99	99.1 ± 1.32
Y-intercept	42.246 ± 0.337	42.060 ± 0.39	nd	nd	nd	37.743 ± 1.15	37.114 ± 0.37	nd	37.281 ± 0.21	37.030 ± 0.63
Value of fit (R^2^)	0.996 ± 0.001	0.998 ± 0.001	nd	nd	nd	0.998 ± 0.002	0.995 ± 0.006	nd	0.997 ± 0.003	0.994 ± 0.003

The experiments was assessed in duplicate over three independent experiments (n = 6). DNA from healthy roots and leaves.

^(a)^*Cq*, quantification cycle; SD, standard deviation; CV%, interassay coefficient of variation: CV% = SD/*Cq* × 100.

*Single sample amplification in only one experiment.

na, not amplified.

nd, not determined.

**Table 2:** Limit of detection of ‘*Candidatus* Phytoplasma solani’ *tuf* gene estimated by qPCR-HRM standard curve performance of: infected Periwinkle leaf by ‘*Ca*. P. solani’ for the 19–25 and P7 isolates; infected grapevine S-y5/4 sample extracted from root and leaf tissue.

	Infected Periwinkle leaf		S-y5/4 sample	
	19–25	P7	Root	Leaf
**DNA dilution (ng/μL)**	***Cq*** **mean** **±** ***SD*** **(*CV%*)**^**(*a*)**^			
1	21.81 ± 0.41 (1.88)	22.43 ± 0.13 (0.57)	30.12 ± 0.57 (1.89)	28.92 ± 0.51 (1.8)
1 × 10^−1^	25.32 ± 0.36 (1.42)	25.36 ± 0.17 (0.67)	33.05 ± 0.37 (1.11)	31.95 ± 0.37 (1.16)
1 × 10^−2^	28.35 ± 0.33 (1.16)	29.09 ± 0.14 (0.48)	36.51 ± 0.58 (1.58)	35.37 ± 0.68 (1.92)
1 × 10^−3^	32.19 ± 0.23 (0.71)	32.56 ± 0.27 (0.82)	na	36.1*
1 × 10^−4^	35.28 ± 0.36 (1.01)	35.93 ± 0.13 (0.36)	nd	nd
1 × 10^−5^	37.2*	na	nd	nd
**Statistics of standard curve performance** ***mean*** **±** **SD**				
Slope	−3.414 ± 0.039	−3.363 ± 0.031	−2.689 ± 0.016	−3.117 ± 0.017
Efficiency	96.3 ± 1.55	98.28 ± 1.36	135.2 ± 1.121	109.3 ± 0.854
Y-intercept	20.045 ± 1.14	21.057 ± 0.196	30.4563 ± 0.718	27.456 ± 1.218
Value of fit (R^2^)	0.996 ± 0.001	0.998 + 0.001	0.98 + 0.012	0.993 + 0.005

Five microlitres of DNA template were used per individual PCR reaction. The experiments was assessed in duplicate over three independent experiments (n = 6).

^(a)^*Cq*, quantification cycle; SD, standard deviation; CV%, inter-assay coefficient of variation: CV% = SD/*Cq* × 100.

*Single sample amplification in only one experiment.

na, not amplified.

nd, not determined.

**Table 3:** Quantification cycle (*Cq*) data collected for different first-step PCR cycle numbers calculated according to different starting DNA concentrations extracted from Periwinkle infected by ‘*Candidatus* Phytoplasma solani’ P7 isolate and root sample from BN symptomatic plant S-y5/4.

Sample	DNA concentration	*Cq* according to cycle no. during first step of PCR					
		10	15	20	25	30	35
P7	1	22.0 ± 0.8	17.8 ± 1.5	13.9 ± 1.3	9.0 ± 0.9	4.7 ± 0.9	2.4 ± 0.9
	1 × 10^−1^	24.9 ± 1.2	21.0 ± 1.9	19.2 ± 1.9	17.2 ± 1.1	10.8 ± 1.2	8.2 ± 1.2
	1 × 10^−2^	28.3 ± 2.1	25.3 ± 0.8	20.4 ± 0.9	19.6 ± 1.3	11.3 ± 1.3	10.8 ± 1.4
	1 × 10^−3^	30.4 ± 0.9	28.7 ± 1.5	27.4 ± 1.1	25.3 ± 1.5	23.2 ± 0.9	20.3 ± 1.8
S-y5/4	1 × 10^−1^	33.3 ± 1.5	33.1 ± 2.1	32.3 ± 1.2	31.0 ± 0.9	31.8 ± 1.1	30.2 ± 0.9
	1 × 10^−2^	35.6 ± 1.4	35.2 ± 1.1	34.9 ± 1.5	34.0 ± 2.4	33.9 ± 1.3	33.1 ± 1.7

*Cq* data are from two technical replicates, repeated twice (n = 4).

Data are means ± standard deviation.

**Figure 1:**
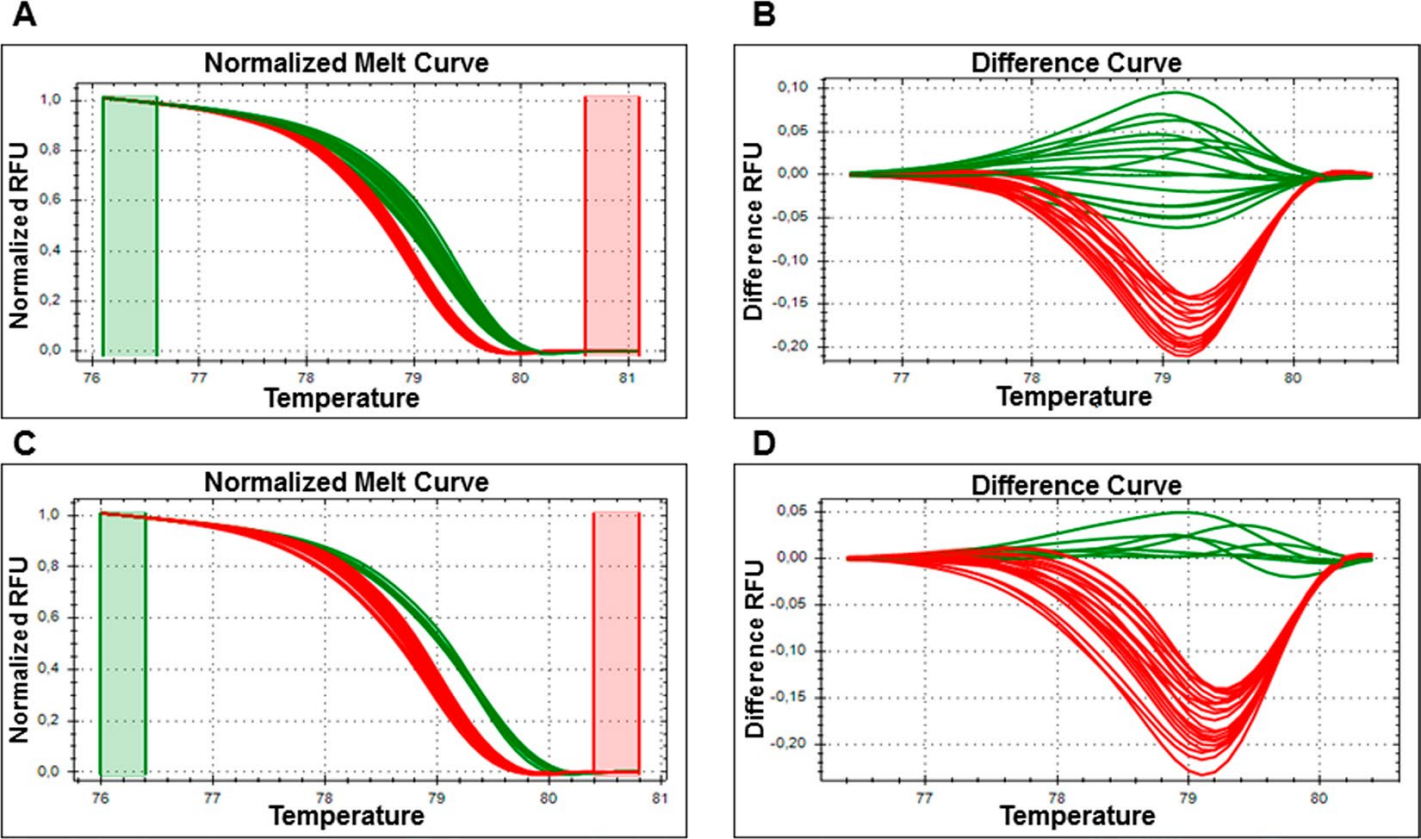
qPCR-high-resolution melting (HRM) analysis to discriminate between *tuf*-type a and *tuf*-type b1. (**A**,**B**) qPCR-HRM analysis of 10-fold serial dilutions of DNA from leaf tissue (1 to 1 × 10^−4^ ng/μL) and per PCR purified fragment (from 5 × 10^−5^ to 5 × 10^−9^ ng/reaction; corresponded to 4.01 × 10^5^ to 4.01 *tuf* PCR fragment copies/reaction) of 19–25 (*tuf*-type a) and P7 (*tuf*-type b1) calibrators. (**C**,**D**) qPCR-HRM analysis of DNA extracted from leaf tissue of symptomatic plants used as control (see Table 4). Two typical genotyping patterns as normalised melting curves (**A**,**C**) and normalised difference plots (**B**,**D**) are shown. Different colours indicate distinct clusters (green, *tuf*-type; red, *tuf-*type b1). RFU: relative fluorescence units.

**Figure 2:**
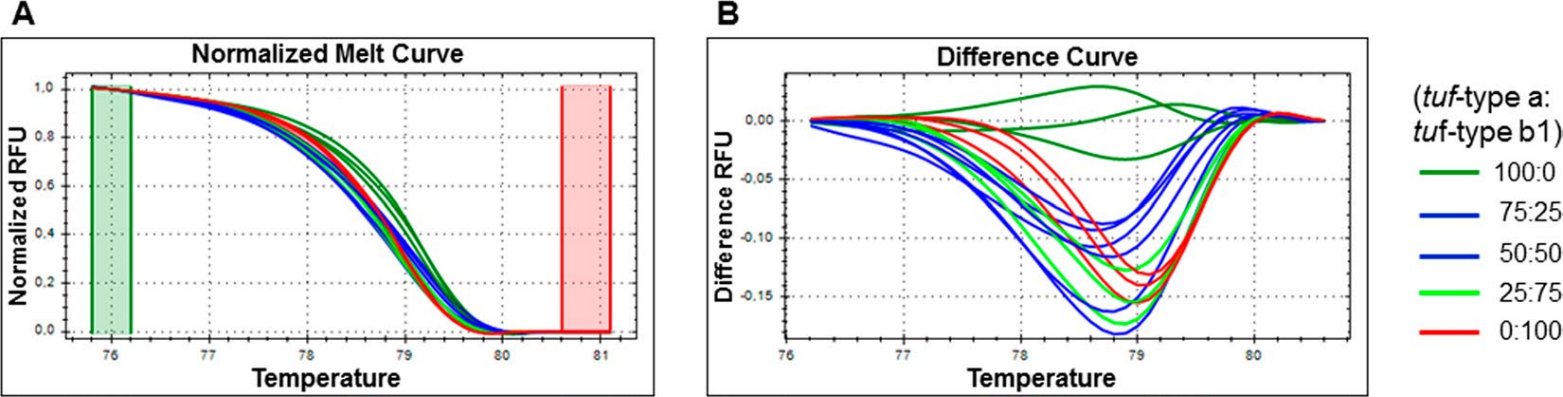
Artificial samples created by mixing the DNA obtained from Periwinkle infected by ‘*Candidatus* Phytoplasma solani’, for the 19–25 (*tuf*-type a) and P7 (t*uf*-type b1) isolates used as calibrators. qPCR-HRM analysis of different concentrations of *tuf*-type a: *tuf*-type b1 as 100:0, 25:75, 50:50, 75:25 and 0:100. Typical genotyping patterns as normalised melting curves (**A**) and normalised difference plots. (**B**) Different colours indicate distinct clusters. RFU: relative fluorescence units.

The HRM assay applied to the dilutions of the calibrator samples (i.e., P7, 19–25) and the PCR purified fragment (Fig. 1A,B), as well as the control samples from the BN symptomatic leaves (Table 4 and Fig. 1C,D), distinguished two different clusters, in agreement with the PCR-RFLP assays^29^ (data not shown). When the artificial samples created by mixing the P7 and 19–25 calibrator samples (representative of two *tuf* types) were analysed by qHMR, an additional cluster was shown that was different from that obtained when these were analysed as 100% calibrator samples for P7 and 19–25. (Fig. 2).

**Table 4:** ‘*Candidatus* Phytoplasma solani’ detection carried out according to qPCR-HRM and nested-qPCR-HRM assays on DNA extracted from root and leaf (control) tissues from BN symptomatic and recovered grapevines.

No.	Plant code	Positive qPCR-HRM assay						Positive to nested qPCR-HRM assay		Positive to TaqMan assay^17^	Positive to conventional nested PCR assay^31^
		Roots			Leaves			Roots			
		*Cq*	*Tuf*-type (copies/5 ng DNA)	*Tuf* type	*Cq*	*Tuf* type (copies/5 ng DNA)	*Tuf*-type	*Cq*	*Tuf* type	*Cq*	+/−
**Symptomatic**											
1	S-y1/2	−	−	−	na	na	na	31.8 ± 0.15	b1	−	−
2	S-y1/3	−	−	−	31.8 ± 0.47	573 ± 56.1	b1	−	−	−	−
3	S-y1/4	−	−	−	28.7 ± 0.32	4120 ± 203.2	b1	30.4 ± 0.16	b1	−	−
4	S-y1/5	−	−	−	29.7 ± 0.20	1943 ± 254.2	b1	31.2 ± 0.2	b1	36.4 ± 0.3	−
5	S-y1/6	−	−	−	na	na	na	−	−	−	−
6	S-y1/8	34.7 ± 0.11	82.3 ± 15.3	b1	na	na	na	27.6 ± 0.32	b1	33.5 ± 0.02	−
7	S-y1/10	−	−	−	na	na	na	28.5 ± 0.29	b1	−	−
8	S-y2/1	−	−	−	na	na	na	−	−	−	−
9	S-y2/4	34.2 ± 0.22	102.3 ± 18.4	a	26.8 ± 0.47	15032 ± 920.0	a	28.2 ± 0.20	a	32.3 ± 0.02	−
10	S-y2/5	−	−	−	na	na	na	−	−	−	−
11	S-y2/6	−	−	−	30.1 ± 0.29	1432 ± 181.0	b1	−	−	−	−
12	S-y3/1	−	−	−	29.2 ± 0.32	3130.4 ± 187.2	b1	−	−	−	−
13	S-y3/2	−	−	−	na	na	na	−	−	−	−
14	S-y3/3	−	−	−	na	na	na	31.1 ± 0.3	b1	−	−
15	S-y3/4	−	−	−	na	na	na	−	−	−	−
16	S-y4/1	−	−	−	Na	na	na	30.6 ± 0.42	b1	−	−
17	S-y4/2	−	−	−	29.3 ± 0.32	2604.1 ± 231.1	b1	−	−	−	−
18	S-y4/3	−	−	−	na	na	na	−	−	−	−
19	S-y4/4	34.5 ± 0.71	83.9 ± 21.0	a	31.2 ± 0.41	902.2 ± 164.3	a	26.3 ± 0.40	a	34.7 ± 0.4	−
20	S-y4/5	−	−	−	na	na	na	31.8 ± 0.22	b1	−	−
21	S-y4/9	−	−	−	na	na	na	−	−	−	−
22	S-y4/10	−	−	−	31.2 ± 0.35	834.4 ± 107	b1	31.2 ± 0.31	b1	−	−
23	S-y5/2	−	−	−	na	na	na	26.6 ± 0.72	b1	−	−
24	S-y5/3	−	−	−	na	na	na	31.3 ± 0.40	b1	−	−
25	S-y5/4	31.5 ± 0.28	684.2 ± 97.0	b1	29.2 ± 0.41	2931.5 ± 282.6	b1	25.4 ± 0.22	b1	30.4 ± 0.8	+
26	S-y5/5	33.7 ± 0.35	162.7 ± 19.5	b1	28.8 ± 0.13	4231.1 ± 232.0	b1	28.2 ± 0.38	b1	31.6 ± 0.3	−
27	S-y5/6	−	−	−	29.2 ± 0.22	3100.4 ± 143.3	b1	30.7 ± 0.35	b1	−	−
28	S-y5/7	34.4 ± 0.24	88.5 ± 16.3	b1	na	na	na	26.4 ± 0.38	b1	33.4 ± 0.4	+
**Total symptomatic**				**6**	**12**				**17**	**7**	**2**
**Recovered**											
1	R-y1/2	−	−	−	na	na	na	35.4 ± 0.21	a	−	−
2	R-y1/4	−	−	−	na	na	na	30.2 ± 0.40	b1	−	−
3	R-y1/5	−	−	−	na	na	na	−	−	−	−
4	R-y1/6	−	−	−	na	na	na	−	−	−	−
5	R-y1/11	−	−	−	na	na	na	−	−	−	−
6	R-y2/1	−	−	−	na	na	na	30.3 ± 0.21	b1	−	−
7	R-y2/2	−	−	−	na	na	na	−	−	−	−
8	R-y2/3	−	−	−	na	na	na	−	−	−	−
9	R-y2/4	−	−	−	na	na	na	27.7 ± 0.31	b1	−	−
10	R-y2/5	34.6 ± 0.61	79.1 ± 18.7	b1	na	na	na	28.9 ± 0.24	b1	32.5 ± 0.32	−
11	R-y2/7	−	−	−	na	na	na	−	−	−	−
12	R-y2/10	−	−	−	na	na	na	31.0 ± 0.21	b1	−	−
13	R-y3/1	−	−	−	na	na	na	−	−	−	−
14	R-y3/4	−	−	−	na	na	na	32.7 ± 0.41	a	−	−
15	R-y3/6	−	−	−	na	na	na	−	−	−	−
16	R-y3/8	−	−	−	na	na	na	−	−	−	−
17	R-y4/1	−	−	−	na	na	na	−	−	−	−
18	R-y4/8	35.4 ± 0.32	44 ± 12.3	a	na	na	na	31.3 ± 0.41	a	34.1 ± 0.2	+
19	R-y4/4	−	−	−	na	na	na	30.3 ± 0.34	a	−	−
20	R-y4/5	−	−	−	na	na	na	29.6 ± 0.32	b1	−	−
21	R-y4/6	−	−	−	na	na	na	−	−	−	−
22	R-y4/3	−	−	−	na	na	na	−	−	−	−
23	R-y4/9	−	−	−	na	na	na	33.3 + 0.31	b1	−	−
24	R-y5/1	−	−	−	na	na	na	−	−	−	−
25	R-y5/2	−	−	−	na	na	na	30.8 ± 0.42	b1	−	−
26	R-y5/8	−	−	−	na	na	na	−	−	−	−
27	R-y5/9	−	−	−	na	na	na	−	−	−	−
**Total recovered**				**2**	**−**				**12**	**2**	**1**
**Asymptomatic**											
1	A1	−	−	−	na	na	na	−	−	−	−
2	A3	−	−	−	na	na	na	−	−	−	−
3	A4	−	−	−	na	na	na	−	−	−	−
4	A5	−	−	−	na	na	na	−	−	−	−
5	A7	−	−	−	na	na	na	−	−	−	−
6	A9	−	−	−	na	na	na	−	−	−	−
7	A10	−	−	−	na	na	na	−	−	−	−
8	A11	−	−	−	na	na	na	−	−	−	−
**Total asymptomatic**				**0**	**0**				**0**	**0**	**0**

The results obtained according to TaqMan fluorogenic exonuclease probe^17^ and nested PCR^31^, were also shown. Data are for two technical replicates from three independent experiments (n = 6).

Data are means ± standard deviation.

*Cq*, quantification cycle.

Plant code: S, symptomatic; R, recovered; A, asymptomatic; y1, 2, 3, 4, 5, years of symptomatic or recovered condition; /number, plant number; na, not analysed.

**Figure 3:**
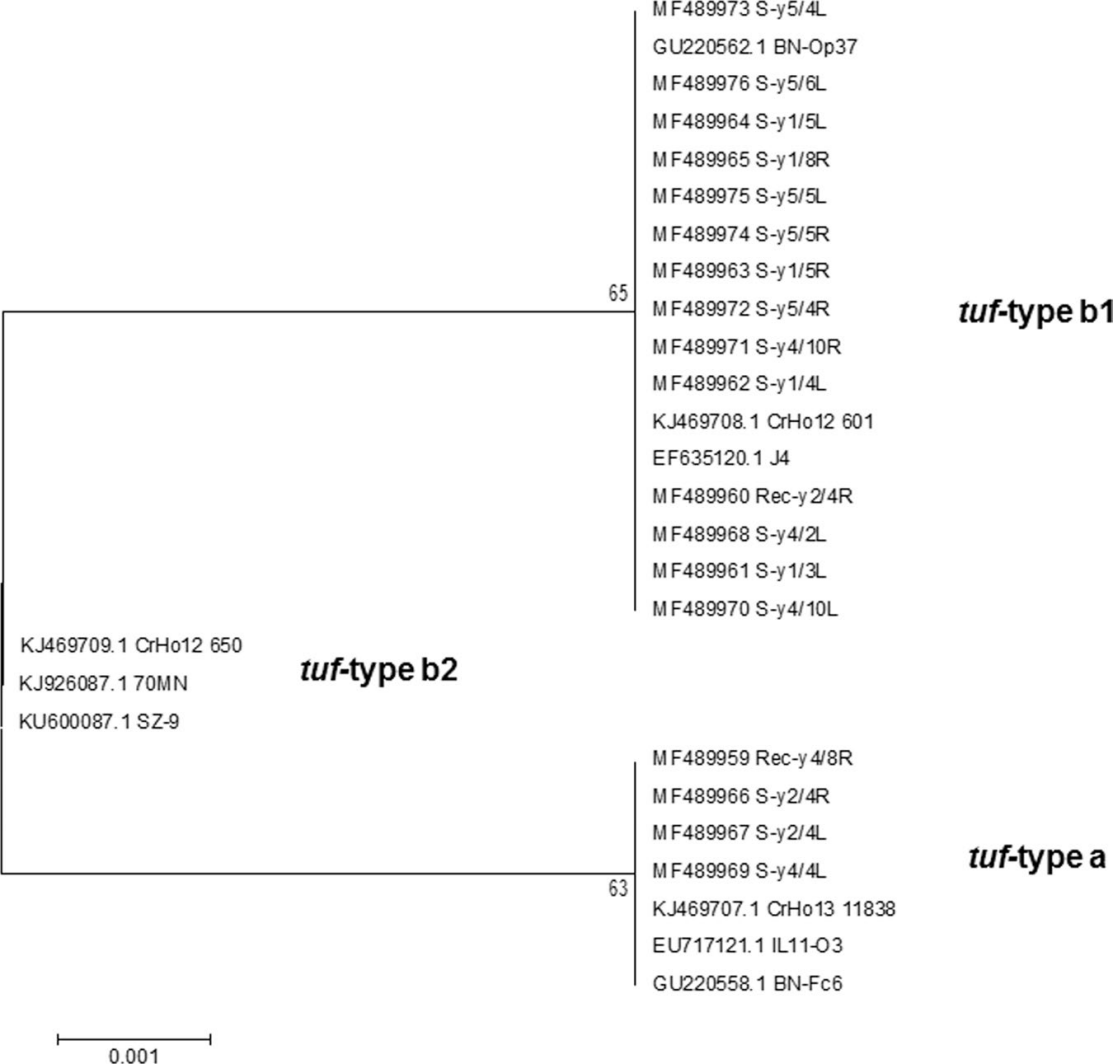
Phylogenetic tree of the *tuf* type sequences from the *Candidatus* Phytoplasma isolates. The *tuf* gene related to isolates selected from symptomatic and recovered plants, showing the relationships among the NCBI sequences selected as references. As reference the following were selected: isolates CrHo13_1183 from *H*. *obsoletus* (NCBI accession No. KJ469707.1), IL11-O3 from grapevine (Croatia; EU717121.1) and *BN-Fc6* from grapevine (Italy; GU220558.1), which were identified as *tuf*-type a; isolates BN-Op37 from grapevine (Italy; GU220562), J4 from grapevine (Croatia; EF635120) and strain CrHo12_601 from *H*. *obsoletus* (Austria), which were identified as *tuf*-type b1; isolates SZ-9 from *Salvia miltiorrhiza* (China; KU600087), 70MN from grapevine (Montenegro; KJ926087) and CrHo12_650 from *H*. *obsoletus* (Austria; KJ469709), which were identified as *tuf*-type b1.

Sequence analysis of the PCR amplicons indicated that the ‘*Ca*. *P*. solani’ isolates R-y4/8R, S-y2/4R, S-y2/4L, S-y4/2L and S-y4/4L clustered with the reference sequences of *tuf*-type a. The isolates R-y2/4R, S-y1/3L, S-y1/4L, S-y1/5R, S-y1/5L, S-y1/8R, S-y4/10L, S-y4/10R, S-y5/4R, S-y5/4L, S-y5/5R, S-y5/5L and S-y5/6L clustered with the reference sequences of *tuf*-type b1. No isolates clustered with reference sequences of t*uf* type b2 (Fig. 3). All of the nucleotide sequences have been deposited in the NCBI GenBank database, with accession numbers from MF489959 to MF489976.

### Detection and characterisation of ‘*Ca*. P. solani’ on grapevine roots

The qPCR-HMR assay detected ‘*Ca*. P. solani’ in six root samples from 28 symptomatic grapevines (21.4%). The nested-qPCR-HMR assay detected ‘*Ca*. P. solani’ in 17 root samples out of the 28 symptomatic grapevines (60.7%) (Table 4). ‘*Ca*. P. solani’ was detected in all of the root samples from plants that had shown symptoms for >5 years, and in 71.4%, 25.0%, 25.0% and 57.1% of the root samples from plants that had been symptomatic for 1, 2, 3 and 4 years (Table 4). ‘*Ca*. P. solani’ was not detected in the roots of the asymptomatic plants (Table 4). ‘*Ca*. P. solani’ *tuf* types were the same in root and leaf tissues tested from the same plant (Table 4). Moreover, the qPCR-HMR assay detected ‘*Ca*. P. solani’ in two root samples out of 27 recovered plants (7.4%). The nested-qPCR-HMR assay detected ‘*Ca*. P. solani’ in 12 root samples (44.4%) (Table 4). ‘*Ca*. P. solani’ was detected in root samples from plants recovered from 1 year (40.0%), 2 years (57.1%), 3 years (25.0%), 4 years (57.1%) and 5 years (25.0%), respectively (Table 4).

The estimated copy numbers of the detected *tuf* gene ranged from means of 82.3 to 604.2 copies/5 ng DNA in the root samples of the symptomatic plants, from means of 44.1 to 79.1 copies/5 ng DNA in the root samples of recovered plants, and these ranged from means of 573 to 15032 copies/5 ng DNA in the symptomatic control leaf samples (Table 4).

**Figure 4:**
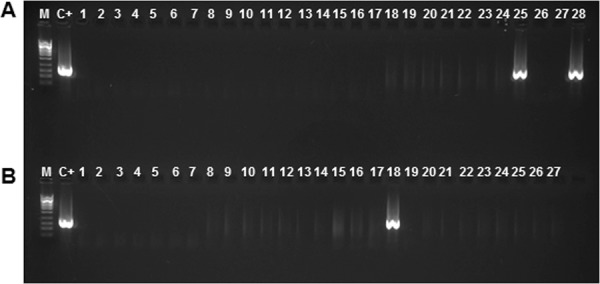
Conventional nested PCR on 2% agarose electrophoretic gels. ‘*Candidatus* Phytoplasma solani’ *tuf* gene detected on root samples collected from BN symptomatic (**A**) and BN recovered (**B**) plants. Amplicon sizes obtained with the primer pair fTuf1/rTuf1 and the nested primer pair fTufy/rTufy. (**A**) Lane 25, S-y5/4; lane 28, S-y5/7. (**B**) Lane 18, R-y4/8 showed an amplicon of ca. 920 bp as the control (C+) P7. M, ladder, 1 kb (New England Biolabs).

The TaqMan qPCR assay^21^ used as the reference tool detected ‘*Ca*. P. solani’ in seven out of 28 symptomatic (25%) and two out of 27 recovered plants (7.4%) (Table 4). On the other hand, the conventional protocols for nested PCR^38^ only detected the phytoplasma in two root samples of the 28 symptomatic plants, and one root sample of the 27 recovered plants (Fig. 4).

**Figure 5:**
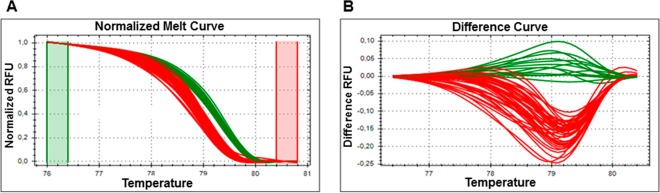
Nested-qPCR-HRM analysis of the DNA extracted from ‘*Candidatus* Phytoplasma solani’ symptomatic and recovered roots samples. Different colours indicate distinct clusters (green, *tuf*-type a; red, *tuf*-type b1). RFU: relative fluorescence units.

The HRM software defined two different clusters that related to these samples: one was linked to the 19–25 calibrator for *tuf*-type a, and the other to the P7 calibrator for *tuf*-type b1 (Fig. 5). Of the 17 samples positive for ‘*Ca*. P. solani’ in the roots of symptomatic plants, 15 clustered with P7 and two with 19–25. Instead, of the 12 positive samples detected in the roots of the recovered plants, eight were linked to P7 and four to 19–25 (Table 4). No other HRM profiles were detected according to the sequence amplicons analysed.

## Discussion

In this study, we report the presence of ‘*Ca*. P solani’ in root samples collected from recovered and BN symptomatic grapevines, where the presence of symptoms had been observed in the canopy of vines for at least 1 year and up to 5 years^28,39^.

The HRM test associated to RT-qPCR technology set-up in this study provides a simple and rapid resource for screening for the presence and relative abundances of *tuf*-type a and *tuf*-type b1 variants of ‘*Ca*. P solani’ in grapevine leaf and root tissues, which can be validated through analysis of the melting curves of the amplicons produced by PCR, without the need for PCR-RFLP^29^ or sequences analysis. These data are further supported by sequence analysis of the PCR amplicons from selected samples. In addition, the qPCR-HMR tests (i.e., represented as a mix of different concentrations of tuf-type a:tuf-type b1), emphasise that these procedures can be used to discriminate between the different *tuf* types, while also simultaneously analysing other molecular variants. However, this study underlines the need to find appropriate DNA template dilutions, in particular for DNA extracted from roots that often included the PCR related to the humic acids in the soil^40^.

*Tuf*-type variants are associated with this BN epidemiology, including their specific association to *H*. *obsoletus* haplotypes and the life strategy of these insect vectors on their plant hosts^3^. The presence of *tuf*-type a and *tuf*-type b1 were detected in the roots from both symptomatic grapevines, where their symptoms were clearly visible on the canopy, and in recovered grapevines, which did not show any leaf symptoms. In contrast, the *tuf*-b2 variant, which is also known as *tuf*-type ab^41^ and has been found only in Austrian vineyards, was not detected here^4^.

The roots and leaves of the same symptomatic plants were shown to be infected by the same ‘*Ca*. P solani’*tuf* type, although the root samples were collected in 2014 and the leaf samples were harvested over the previous 5 years^28,39^. Thus, this study confirms that the phytoplasma in these plants remains the same over time, even across different organs, such as roots and leaves. The prevalence of *tuf*-type b1 in the samples of this study agrees with previous studies of symptomatic grapevine leaves in the Mediterranean basin^39^. A similar result was observed in the roots of recovered plants, although with a relatively higher proportion of positive *tuf*-type a. Further studies that can analyse greater numbers of infected roots from recovered plants are needed to determine whether there is any epidemiological significance associated to this aspect. Although the plants that show recovery from phytoplasma are less likely to become re-infected^13,42^, the presence of a reservoir of ‘*Ca*. P. solani’ in the roots might lead to the reappearance of symptoms in such recovered plants.

The analysis by qPCR-HRM of the root and leaf samples extracted from the same plants showed that the copy numbers of the *tuf* gene were higher in the leaves than in the roots, regardless of type. Therefore, we hypothesise that the main difficulty for detecting this pathogen in grapevine roots will depend on the low phytoplasma concentrations for this organ. The nested-qPCR-HRM improved the phytoplasma detection in roots.

Knowledge of the distribution of phytoplasma across the various plant organs is usually essential for better understanding of the interactions between phytoplasma and their plant host. Typically, phytoplasma diagnosis for grapevines is carried out in a restricted seasonal period, from June to September, when the phytoplasma symptoms are clearly expressed in the leaf tissue. The possibility to test roots and to successfully detect the phytoplasma can expand the time-frame in which phytoplasma testing can be done.

Phytoplasma move within plants through the phloem, from source to sink, and they can pass through sieve-tube elements in phloem tissues^43–45^. Previous studies performed on apple trees on established rootstock that have recovered from apple proliferation have shown that the root systems of these trees remain colonised for the lifetime of the tree^17^.

These data show the presence of ‘*Ca*. P. solani’ in roots from both symptomatic and recovered plants, which suggests that the concentration and location of the pathogen affects the appearance of BN. In addition, the present study shows that all root samples of the plants that were symptomatic for five consecutive previous years were positive for ‘*Ca*. P. solani’; the phytoplasma was detected in 50% of these samples with the qPCR-HRM test, without the following nested qPCR-HRM step. These data demonstrate that the accumulation of phytoplasma in the roots is higher in plants infected over several years. Furthermore, our investigation suggests that the ‘*Ca*. P. solani’ levels in the roots of recovered plants is lower compared to the roots of symptomatic plants; moreover plants recovered over 5 years maintained the phytoplasma in the roots. The potential role of the pathogen in the recovered plant is not completely clear; however, previous studies have shown the induction of defence mechanisms in recovered plants and in asymptomatic parts of infected plants^16,46–48^.

In conclusion, we propose these rapid and easy molecular approaches for detection of ‘*Ca*. P. solani’ *tuf* types in grapevine roots. In particular, we propose the more sensitive nested-qPCR-HRM method, which can be applied to detect phytoplasma at low titres for plant organs such as roots. This might also be useful for the selection of healthy propagation material without the need for the canopy, such as during the winter. These data underline the presence of ‘*Ca*. P. solani’ in roots from both symptomatic and recovered plants, also highlighting that the phytoplasma can persist in the roots irrespective of the presence of disease symptoms on the plant. However, the relatively low number of root samples that were positive to ‘*Ca*. P. solani’ here, as well as the low titres of the phytoplasma detected in the recovered plants compared to the symptomatic plants, indicate that phytoplasma disappearance in grapevine roots is possible. On the other hand, the titre of the pathogen in the roots might affect the balance between appearance and disappearance of symptoms.

## Methods

### Plant root samples

This study was carried out in a vineyard planted with cv. Chardonnay grapevines that covered about 0.6 ha and was located in Montalto Marche (Ascoli Piceno), in central-eastern Italy (42°59′00″N, 13°36′00″E; 513 m a.s.l.). The vineyard had been monitored for ‘*Ca*. P. solani’ over 7 years, from 2008 to 2014^39^. Root samples were collected in September 2014 from plants that were symptomatic (28 plants), recovered (27 plants) for at least 1 year to 5 years, and asymptomatic (eight plants), which had never expressed phyoplasma symptoms (Table 1). Two sub-samples of secondary roots fragments (length, 10–15 cm; diameter, 3–5 mm) were collected from about 20 cm in depth. After washing in tap water, the root sub-samples were put into 0.05% (v/v) Tween 20 in 50-mL tubes (Falcon) and sonicated for 10 min. The roots were rinsed in distilled water, and kept at −20 °C until DNA extraction.

### DNA extraction

Total DNA was extracted from roots using the cetyl trimethyl ammonium bromide (CTAB) procedure^49^. For each sub-sample, 2 g of pooled roots was ground in liquid nitrogen, and 200 mg of the pulverised materials was added to 2-mL microcentrifuge tubes with 1 mL extraction buffer (3% CTAB, 100 mM Tris-HCl, pH 8.0, 20 mM EDTA, 1.4 M NaCl, 2% [w/v] soluble PVP-40), and 1% (w/v) metabisulphite was added. After incubation at 68 °C for 30 min, purification with chloroform/ isoamyl alcohol (24:1), and precipitation with 0.6% isopropanol were conducted. Finally, the DNA was dissolved in 50 μL pure water. The DNA purity and quantity was also determined (BioPhotometer plus; Eppendorf Inc., Westbury, NY, USA) and was assessed on at least 100 ng/µL DNA, with the absorption ratios at 280/260 in the range of 1.6–1.8, and at 260/230 in range of 1.3–2.0. To increase the chance of detection of the phytoplasma, the DNA obtained from the two root sub-samples per plant were merged and analysed.

### Set-up of qPCR-HRM and nested-qPCR-HMR for ‘*Ca*. P. solani’ detection

Detection and characterisation of ‘*Ca*. P. solani’ was carried out in the grapevine root samples using the phytoplasma *tuf* gene, which encodes the translation elongation factor Tu. For testing the reproducibility and sensitivity to detect *tuf*-type variants for both qPCR-HRM and nested-qPCR-HRM protocols, several parameters were evaluated.

**Figure 6:**
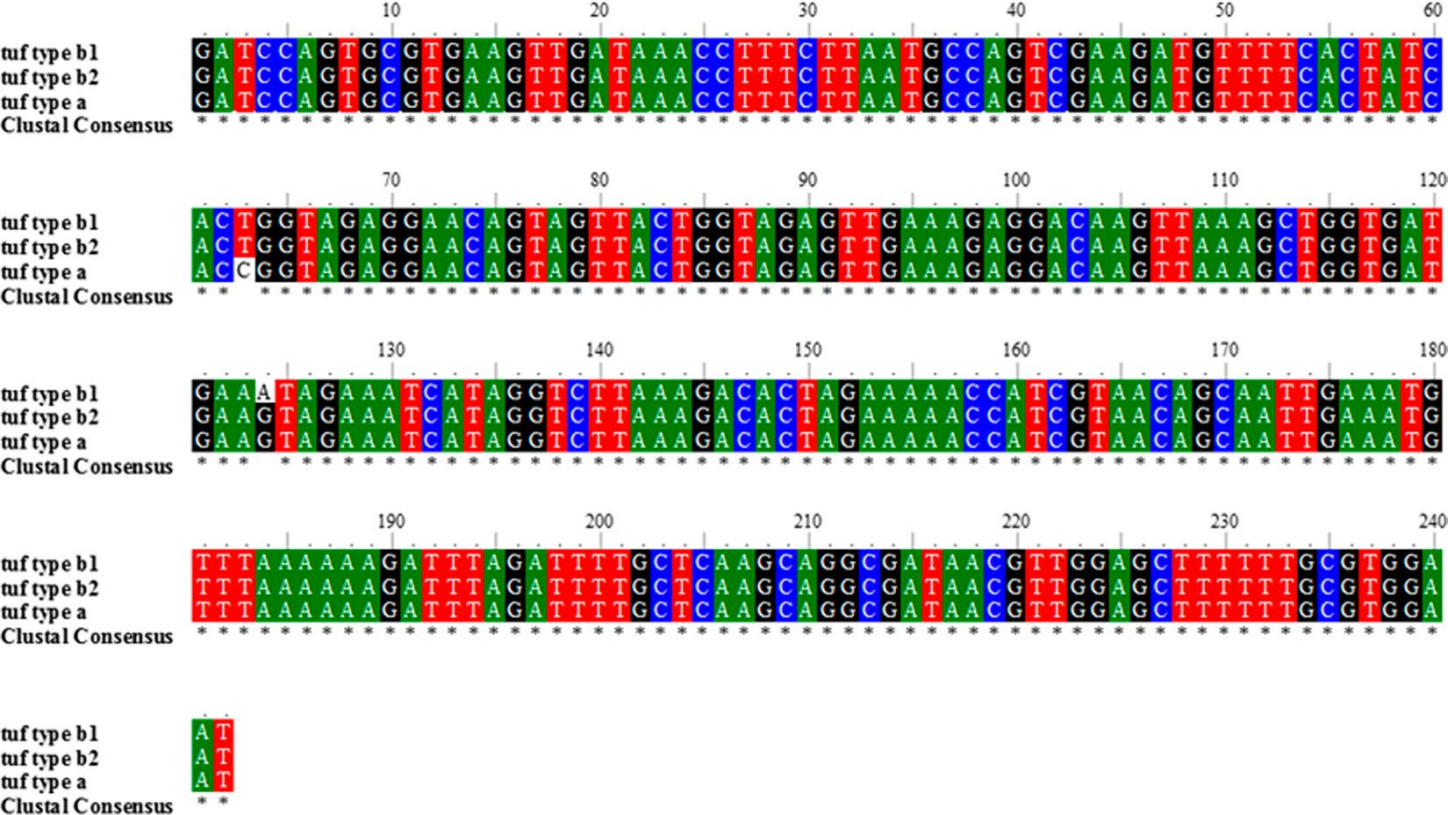
Multiple sequence alignment of representative *tuf* types. The sequence was related to 242 bp PCR fragment amplified by the primers pairs *Tuf*-U/f-r used in this study.

For the primers, the forward Tuf-U/f (5′-GATCCAGTGCGTGAAGTTGA-3′) and reverse Tuf-U/r (5′-ATTCCACGCAACAAAGCTCC-3′) primers were designed using the Primer3 software (http://www.ncbi.nlm.nih.gov/tools/primer-blast/), and the specificity of primers for ‘*Ca*. P. solani’ *tuf* gene sequence was verified using the BLAST programme (http://blast.ncbi.nlm.nih.gov/Blast.cgi). These primers identified a 242-bp amplicon that included the nucleotide substitutions of C → T (position 63; *tuf-*type a → *tuf-*type b1, b2) and A → G (position 124; *tuf-t*ype b1 → *tuf-t*ype a, b2) (Fig. 6). Total plant DNA from the ‘*Ca*. P. solani’ isolates 19–25 (*tuf-*type a) and P7 (*tuf-*type b), used as calibrator samples, was extracted from phytoplasma-inoculated periwinkle plants, kindly provided by Dr. Xavier Foissac (INRA and University of Bordeaux, France). As positive controls, leaf tissue DNA of symptomatic grapevines, previously analysed^28^ were also included (Table 4).

The qPCR inhibitors, the optimal concentration of DNA template, and the limits of quantification (LOQ) and detection (LOD)^50^, estimated from analysis of replicate standard curves, were determined. Firstly, to calculate ‘*Ca* P. solani’ copy number, the purified *tuf* PCR fragments amplified from calibrators by qPCR-HRM were used. The molecular weight (daltons) was determined for a single PCR fragment (http://www.bioinformatics.org/sms2/dna_mw.html), and converting from daltons to nanograms (http://www.unitconversion.org/weight/daltons-to-nanograms-conversion.html). Finally, the number of copies was calculated according to eq. (1):

1 $${\rm{Copy}}\,{\rm{number}}={\rm{quantity}}\,({\rm{ng}})/{\rm{PCR}}\,{\rm{fragment}}\,{\rm{molecular}}\,{\rm{weight}}\,({\rm{ng}}).$$

The LOQ and possible inhibitors of the different matrices (i.e., leaves, roots) with the detection of ‘*Ca*. P. solani’ by qPCR-HRM was investigated, with artificial positive samples generated. In detail, the DNA pool of healthy grapevine root matrix, (500 ng, 100 ng, 75 ng, 25 ng, 5 ng/reaction,) and leaf matrix, (500 ng, 100 ng, 5 ng/reaction) were spiked with the 10-fold serial dilutions purified P7 ‘*Ca* P. solani’ *tuf* PCR fragment (from 5 × 10^−5^ to 5 × 10^−10^ ng/reaction; corresponded to 4.01 × 10^5^ to 4.01 *tuf* PCR fragment copies/reaction). The serial dilution of P7 ‘*Ca* P. solani’ *tuf* PCR fragment alone (positive control) and DNA from healthy roots and leaves (negative control) were included. Moreover, the DNA from infected roots were testing by qPCR-HRM at different concentrations (5 ng, 50 ng, 500 ng/reaction).

The LOD, and discrimination of *tuf-*type variants in qPCR-HRM assays were evaluated according 10-fold serial dilutions (from 1 to 1 × 10^−5^ ng/μL) of DNA from the P7 and 19–25 calibrators, and artificial samples created by mixing DNA from the calibrators P7:19–25 at ratios of 25:75, 50:50 and 75:25 were also analysed. Moreover 10-fold serial dilutions (1 to 1 × 10^−3^ ng/μL) of DNA from positive leaf and root samples included.

The DNA concentration for the nested-qPCR-HRM analysis was selected by testing 1, 1/10, 1/100 and 1/200 dilutions of the PCR products from the first amplification. To determine the optimal PCR cycle number in the first-step of PCR^37^ before the nested-qPCR-HRM analysis, several trials were carried out. The PCR programme was stopped every 5 cycles (from 10–35 cycles) to test the 10-fold serial dilutions of the P7 sample calibrator (1 to 1 × 10^−3^ ng/μL) and the S-y5/4 sample that was positive to ‘*Ca*. P. solani’ (1 × 10^−1^ to 1 × 10^−2^ ng/μL). This experiment was carried out in duplicate and was repeated twice.

For nested qPCR-HRM assays, the HRM reproducibility was estimated using the PCR template of 10-fold serial dilutions (from 1 × 10^−5^ to 1 × 10^−10^ ng/μL) of PCR fragments generated from the calibrators.

The qPCR-HRM and nested qPCR-HRM trials were carried out in duplicate over three independent experiments. Reproducibility of the results was checked by interassay analysis, and the Cq mean was calculated for all of the standard dilutions. The variability are expressed as SD and CV%.

To discriminate between *tuf*-type a and *tuf*-type b using qPCR-HRM, *tuf* gene PCR amplicons of representative ‘*Ca*. P. solani’ isolates were sequenced (Genewiz, Hope End, Takeley, UK). The analysed phytoplasma isolates included: leaf and root tissues from five symptomatic plants (Table 4, S-y1/5, S-y2/4, S-y4/10, S-y5/4, S-y5/5); leaf tissue from five symptomatic plants (Table 4, S-y1/3, S-y1/4, S-y4/2, S-y4/4, S-y5/6); and root tissue from one symptomatic plant (Table 4, S-y1/8) and two recovered plants (Table 4, R-y4/8, R-y2/4). Sequence similarity searches were performed using Blast analysis in NCBI. Multiple sequence alignments were constructed using Clustal_X^51^. Phylogenetic trees were constructed using the Molecular Evolutionary Genetics Analysis (MEGA) programme, version 5.2 (http://www.megasoftware.net/index.html)^52^, according to the neighbour-joining method^53^, with 1,000 bootstrap replicates. Estimates of the average evolutionary divergence over sequence pairs were made using the Maximum Composite Likelihood model for the *tuf* sequences. The average genetic distances among the clades inferred by the phylogenetic analysis were computed according to the Jukes-Cantor model^54^, using the MEGA software.

### Detection and characterisation of ‘*Ca*. P. solani’ on grapevine roots

For qPCR-HRM assays, 5 μL (1 ng/μL) DNA template was used for all of the experiments. For the nested-qPCR-HRM, the DNA extracted from root test samples in the first step was amplified using the fTuf1/rTuf1 primer set, using conventional PCR^38^. For the PCR mix, 10 ng DNA was included in each 20 µL PCR reaction, with 1 mM of each primer, 10 μL 2x EconoTaq Plus Green Master Mix (Lucigen; Tema Ricerca S.r.l., Castenaso, Bologna, Italy). The products from the first amplification were diluted 1/200 in ultrapure water, and 5 μL was used as the DNA template in the nested-qPCR-HMR assays. Finally, all qPCR-HRM and nested-qPCR-HRM amplifications were carried out in a total volume of 14 μL, which in addition to the DNA template described above, contained 7 μL SsoFast EvaGreen Supermix (Bio-Rad Laboratories, Hercules, CA, USA), and 1 μL of the designed primers (1 mM each). The reactions were subjected to the following conditions: initial denaturation step for 3 min at 98 °C, followed by 40 cycles of 20 s denaturation at 98 °C, and 40 s annealing–elongation at 60.5 °C. The final step included the melting curve analyses (0.2 °C step increments; 10 s hold before each acquisition), which were analysed from 70 °C to 95 °C. The quantification of the samples in the qPCR-HRM was performed according to the standard curve previously described. The qPCR-HRM and nested-qPCR-HRM amplifications were both performed using the CFX real-time PCR detection system, and analysed using the ‘High-Resolution Melting analysis software’ (Bio-Rad Laboratories). This software automatically clusters the samples according to their melting profiles and assigns confidence scores to each of the samples. The confidence level threshold for a sample to be included in a cluster was 99.0%. As controls, all of the root samples were subjected to qPCR-HRM and nested-qPCR-HRM, and the performances were compared with the data obtained by applying conventional nested PCR^38^, and RT-PCR using TaqMan fluorogenic exonuclease^21^. The conventional PCR was performed in three independent experiments, and all the qPCR-HRM and nested-qPCR-HRM trials were assessed in duplicate over three independent experiments.
